# Effects of project-based learning on EFL learners’ writing performance

**DOI:** 10.1371/journal.pone.0317518

**Published:** 2025-01-31

**Authors:** Abebaw Andargie, Dawit Amogne, Ebabu Tefera

**Affiliations:** 1 Department of Language Education, College of Education, School of Teacher Education, Bahir Dar University, Bahir Dar, Ethiopia; 2 Department of English Language and Literature, College of Language Studies, Addis Ababa University, Addis Ababa, Ethiopia; Faculty of Electrical Engineering, Computer Science and Information Technology Osijek, CROATIA

## Abstract

Connecting language classrooms with 21st-century skills could be the potential framework for enhancing EFL learners’ performance in writing classes. However, investigating whether project-based learning, as a new field within ELT with unique pedagogical affordances, can enhance learners’ writing skills still needs to be improved in the literature. Accordingly, this study aimed to investigate the impact of project-based learning on EFL learners’ writing performance. It sought to determine whether and to what extent project-based learning could enhance writing skills in an EFL context. The study employed a quasi-experimental design with an interrupted pre-test-post-test time series design with single group participants. Twenty-three third-year EFL undergraduate students enrolled in the Advanced Writing Skills I course were selected using a comprehensive sampling method. An essay writing test and interview were used to gather data. The participants of the study were given a series of three problem-solving essay writing tests before and after the intervention, which employed project-based essay writing instruction. In addition, to discover their attitudes toward the impacts of project-based learning and its applications on the ground, three randomly selected students were interviewed at the end of the intervention. The data collected through the tests were analyzed through a one-way repeated measure ANOVA; narration was also used to analyze the qualitative data gathered through interviews. Accordingly, the quantitative data suggested that project-based learning significantly enhances EFL learners’ writing performance. Moreover, interview data showed that students felt optimistic about the impact of project-based learning on their writing performance, idea generation, and cooperation among themselves. Therefore, project-based learning is suggested as another method in ELT writing classes because it enhances learners’ writing via idea generation, data collection, organization, cooperation, and general communication skills. As students work on worthwhile projects, its emphasis on real-world applicability and realistic activities can help them become better writers. Hence, teachers can reinforce the relationship between form and purpose by incorporating a variety of genres and collaborative writing to reflect real-world or professional situations.

## Introduction

Writing is a crucial skill for students to succeed academically and professionally. It plays a pivotal role in EFL education as it enables students to express their thoughts, ideas, and emotions effectively in written form. Proficient writing skills empower EFL students to communicate confidently, accurately, and fluently in academic settings and various personal and professional contexts. It is an essential language skill that strengthens vocabulary acquisition, enhances grammatical accuracy, fosters critical thinking, and nurtures creativity.

In EFL contexts, according to [1] writing skills are complex constructs but crucial abilities that involve combining words and sentences coherently to express ideas. The development of the skill can be positively or negatively influenced by various factors. Additionally, the complex nature of writing, including organizing ideas, structuring paragraphs, and incorporating appropriate language conventions, presents difficulties for EFL learners. Various studies have also identified and witnessed students’ problems in writing essays. [2] found that students need help generating ideas, vocabulary, and grammatical elements, particularly in English expository essays. [3] highlighted problems in prewriting, coherence, cohesion, lexicon, and mechanics, with planning unfamiliar topics and developing main ideas particularly challenging. [4] identified cognitive, linguistic, and psychological issues in writing argumentative essays, including a need for knowledge of critical features, content, grammar, organization, vocabulary, and fear of making mistakes. These findings underscore the need for targeted support and the implementation of effective strategies to facilitate students’ writing development. Teachers must navigate these challenges and implement effective strategies to facilitate students’ writing development. In addition, according to [5], learning English as a foreign language involves a wide range of disciplines. To improve student participation and engagement and lessen test anxiety, various interactive platform types have been implemented in EFL learning settings. Beyond the theories, practical instruction in EFL classrooms is vital to equipping students with the writing skills required for academic success and future career opportunities. Project-based learning has emerged as a practical approach for enhancing EFL students’ writing skills.

The ideas of early 20th-century educator John Dewey, who advocated "learning by doing," are where project-based learning originated. William Heard Kilpatrick refined this strategy further by introducing a significant essay called "The Project Method," which emphasized student-centred projects and reinforced the importance of active learning in school [6]. As described by [7] project-based learning is an approach that emphasizes teaching by having students engage in investigations. It allows them to be fully involved and take the initiative. In addition, [8] stated that project-based learning is an extended effort that comprises a range of individual and group activities, such as establishing research questions and plans and carrying them out via practical research, which entails gathering and documenting data verbally and in writing. Hence, it is essentially a collaborative learning approach where students study and solve real-world issues to produce a concrete product or solution.

In language classrooms, project-based learning is a teaching approach that emphasizes applying language skills in real-world contexts [9]. It is particularly effective in developing students’ life skills, such as communication, mutual respect, and self-regulation [10]. These studies highlight the benefits of project-based language learning in promoting practical language use and the development of essential life skills. Project-based learning provides authentic contexts and meaningful tasks that prompt students to apply their writing skills in real-world situations. This enhances the relevance and practicality of their writing abilities. Through collaborative writing projects, students develop teamwork, communication, and interpersonal skills. Working together fosters communication, increases motivation, and promotes active participation. It also promotes critical thinking and problem-solving skills as students research information and make informed decisions while completing their writing projects. Hence, it allows students to explore various genres and formats, resulting in more prosperous and engaging writing.

Furthermore, navigating the all-around advantages of project-based learning, this study aims to investigate the effects of project-based learning on EFL learners’ writing performance. It intends to contribute to the existing literature on effective writing instruction and provide insights for educators on innovative teaching practices that can enhance students’ writing proficiency.

As the study of [11] clearly states, writing well in English becomes even more crucial as students advance in their academic careers. To complete their degree requirements, higher education students must produce various academic projects, including papers, articles, summaries, and in-depth research theses. That is why writing courses are compulsory subjects in Ethiopian higher education institutions. Writing is designed for high-quality training programs in the English curriculum to improve students’ academic writing skills and enable them to use the skill in their academic training and general and future career development. Although there was an emerging inclination towards learner-centred approaches, the educational landscape is dominated by teacher-led methods, and writing guidelines suggest that students must be more involved in their education and acquire the ability to write in multiple genres. Various interactive platform types have been used in the context of teaching English as a foreign language (EFL) to increase student interest, engagement, and learning satisfaction [12].

For many years, educators have been concerned with improving writing abilities among EFL learners. Numerous studies on writing proficiency in Ethiopian universities reveal remarkable barriers. According to [13], university students in Ethiopia need to improve in all areas of writing, including content, organization, grammar, vocabulary, and mechanics. In the words of [14], although the majority of learners acknowledge the relevance of writing skills, it has been discovered that undergraduate English programs do not adequately educate writing to help students improve their talents, and teachers frequently default to product-oriented strategies instead of applying the process approach to writing instruction. The above findings highlight the need for reforms in Ethiopian higher education’s writing instruction. Hence, there is a pressing need for innovative instructional strategies that improve students’ writing performance.

### Statement of the problem

When the researcher taught ELT writing courses in a higher education institution, it became evident that there needed to be more interest in writing classes at the school where the study was done; however, the expected performance of students needed to be better. Along with the researcher, many educators expressed dissatisfaction with their students’ poor writing performance in their EFL classes. They stated that many writing tasks needed to be more engaging and practical. To be more specific, the researcher experienced that in ELT writing classes; only some students did tasks appropriately. The majority of them were not involved in the lessons, which needed active participation and cooperation among students, and their written work could have been better. When it comes to capturing students’ attention, involving them, and developing the skills needed for effective writing, traditional teaching approaches frequently need to be revised.

Regarding the positive impact of project-based learning in EFL settings, several studies have forwarded their thoughts and recommendations. These studies showed that project-based learning significantly improves EFL learners ’ writing skills at different levels and in different contexts. For instance, [15] demonstrated that project-based learning enhanced Iranian EFL learners ’ comparison and contrasted paragraph writing skills compared to traditional instruction. In the same fashion, the study by [16] indicated that it had a tremendous effect on Iranian EFL learners ’ writing ability in a collaborative environment. The study by [17] further reported that project-based learning significantly improved junior high school students’ writing skills in Indonesia while also promoting critical thinking, communication, and creativity. In recent years, [18] confirmed project-based learning’s positive effect on Indonesian junior high school students’ descriptive writing skills, with a large effect size. These studies collectively suggest that project-based learning is a practical approach to improving EFL learners’ writing skills across different contexts and genres.

However, studies that investigated the specific effects of project-based learning on EFL learners’ writing performance are limited, and there is also a paucity of empirical evidence detailing how project-based learning influences various dimensions of writing proficiency, such as coherence, grammar, vocabulary, and overall fluency, among EFL learners. In addition, little is known about the contextual elements such as cultural background, language ability, and classroom setting. The local context in which the current study is conducted should have been considered in the studies above. In the current study area, project-based learning gets less attention and privilege in EFL classrooms, especially in writing classes. As far as the researcher’s knowledge is concerned, studies have yet to be conducted on the area in the Ethiopian context. In addition, most of the studies conducted on the application of project-based learning used an experimental design in which control and experimental groups existed. Hence, conducting a study on the effect of project-based learning on EFL learners’ writing skills in this local context and through another design was fundamental to filling the research gap in the area and encouraging stakeholders to incorporate this instruction into the curriculum.

Therefore, considering the gaps in the area and the urgency of the problem, this study sought to investigate the effects of project-based learning on EFL learners’ writing performance by employing a single-group interrupted time series design. It aimed to address the gaps and to determine whether and to what extent project-based learning could enhance writing skills in an EFL context through a systematic investigation. This research would make a significant contribution to the field of EFL teaching and offer helpful suggestions to EFL teachers who want to enhance writing instruction by utilizing cutting-edge pedagogical strategies.

### Research question

Considering the already heated discussion on the effectiveness of project-based learning in writing class and with the attention to filling the current gap of knowledge on project-based learning and contributing to the ongoing discourse on effective teaching practices in EFL education and providing practical insights for educators, curriculum developers, and policymakers seeking to improve writing instruction for EFL learners, this study was designed to answer the following research question:

Does project-based learning significantly improve EFL students’ writing performance?

## Literature review

The ideas of early 20th-century educator John Dewey, who advocated ’learning by doing,’ are where project-based learning got its origins. William Heard Kilpatrick refined this strategy further by introducing a significant essay called ’The Project Method,’ which emphasized student-centred projects and reinforced the importance of active learning in school [7]. As described by [9], project-based learning is an extended effort that comprises a range of individual and group activities, such as establishing research questions and plans and carrying them out via practical or document research, which entails gathering and documenting data verbally and in writing. So, it is essentially a collaborative learning approach where students, as a community, study and solve real-world issues to produce a concrete product or solution. In addition, [8] stated that project-based learning is an approach that emphasizes instructing by having students engaged in investigations, which allows students to be fully engaged and take initiative. Thus, in project-based learning, learners engage with their peers in investigating and solving real-life problems.

The lack of a universally agreed-upon definition, according to [19] has led to a variety of project-based learning research and development initiatives. Nonetheless, a content analysis of the aforementioned noteworthy definitions demonstrates that they are complimentary due to specific shared characteristics. These attributes are in complete harmony with the foundational ideas of constructivism and the drive to develop PBL. Moreover, numerous writers have explained the essential elements and stages of project-based learning. For instance, [20–22] have compiled lists of essential project design elements, including a challenging problem or question, sustained inquiry, authenticity, student voice and choice, reflection, critique, and revision, and a final public product. In addition, [23] outlined project-based learning steps such as preparation, topic definition and selection, project layout, project implementation, presentation, and evaluation. Accordingly, project-based learning has unique elements and steps that should be used in its application.

Project-based learning aims to enhance students’ learning experiences by engaging them in real-world projects that are designed to address authentic, complex problems or challenges that require critical thinking, collaboration, and creativity. Through this instruction, EFL students can develop and improve their writing skills in a meaningful and practical way. Various studies support this idea. For example, a study by many scholars found that project-based learning not only boosts students’ writing skills but also makes the classroom more vibrant with engaging activities. Furthermore, project-based learning can create a better learning situation for EFL students as it becomes real with various exciting activities [24].

A variety of studies, each with its unique focus, have demonstrated the effectiveness of project-based writing instruction in enhancing students’ writing skills. [25] found that project work can significantly improve students’ writing skills, while [26] highlighted its role in developing students’ ability and creativity in writing narrative texts. [27] further supported these findings, reporting that project-based learning can boost students’ confidence in writing and improve their communication skills. [28] extended these findings to the field of social work, showing how project-based learning can be used to enhance writing skills in this context. Therefore, previous research studies in project-based learning and writing instruction reached the same successful conclusions.

## Materials and methods

### Design

This study tried to investigate the impacts of using project-based learning on EFL students’ writing performance. The research design of the study was quasi-experimental and employed a time series design with single-group participants. For this study, the participants were given a series of three problem/solution essay writing pretests before the intervention, project-based essay writing instruction, and other three similar series of problem/solution essay writing posttests. Quasi-experimental designs, such as the pretest-posttest design, are commonly used in various fields. These designs can be used to demonstrate causality between an intervention and an outcome, particularly in natural settings where randomization is not feasible [29]. In these cases, the researchers utilized a quasi-experimental design to study the effects of project-based learning on EFL learners’ writing skills.

### Participants

In this study, a total of twenty-three third-year EFL learners enrolled in the Advanced Writing Skills I course in the Department of English Language and Literature at Debre Markos University were selected using a comprehensive sampling method as the study aimed to fully explore the experiences of all participants involved in the project. During their time at university, the students had already completed courses in Communicative English Skills, Basic Writing Skills, and Intermediate Writing Skills. Before these university classes, they had studied English from early childhood through university.

### Tools

To achieve the study’s desired objective, the researcher gathered both quantitative and qualitative data. The instruments utilized for this research were tests and interviews.

#### Tests

Essay writing tests have been used as a valuable tool for data collection in various academic or research contexts. Many scholars used such tests to explore students’ writing performance. In the current study, essay writing tests that comprised both pretests and posttests were used to gather data on EFL learners’ writing performance. Three consecutive problem-solving essay writing pretests were given to understand the students’ existing writing performance before the intervention; again, three other consecutive essay writing posttests were given to discover the effects of the intervention. Considering the students’ level and background knowledge, the researcher has prepared these tests. The face and content validity of these tests was ensured through a rigorous process, with two supervisors and two PhD candidates in TEFL from Bahir Dar University meticulously checking the item value, clarity of instructions, level of complexity, and time given, leaving no room for doubt about the quality of the study. Two EFL teachers who have taught the course marked the test scores using the standardized British Council English Language Testing System (IELTS) writing task descriptors. The reliabilities were calculated, and the values were 0.80, which implied that the tests were reliable in measuring the students’ writing skills.

#### Interview

A semi-structured interview was applied to get more in-depth information on the application of project-based learning in enhancing learners’ writing performance and learners’ attitudes towards it. Accordingly, one student from each of the four project groups, a total of four students was selected randomly and interviewed face-to-face for about 10 minutes. A balanced understanding of the collective experience was provided by choosing one student from each group, ensuring that perspectives from all groups were represented. Focusing on fewer interview subjects allowed the researcher to probe their experiences more thoroughly, resulting in rich, in-depth answers that strengthened the qualitative aspect of the study. The interview was conducted at the university’s HDP Room in Building 24 at Debre Markos University. To make them express their feelings well and freely, the purpose of the interview and the interview items were explained to the participants before the interview. Their responses were recorded, transcribed, and finally analyzed through narration.

### Procedures and data collection

The study used procedures to achieve the desired objective while applying the intervention and collecting data. It focused on essay writing among third-year EFL students for six weeks, from 24 October to 9 December 2023. Before the data were gathered and the intervention was made, the researchers communicated with the academic officers and research and ethical committee. They got permission from that committee to conduct the study. Hence, ethical consideration was maintained by respecting the privacy and other rights of the participants involved in the study were provided with detailed information about the nature and purpose of the study; their voluntary agreement to participate was documented, and informed consent was obtained from them. The confidentiality and welfare of the participants were prioritized throughout the research process. In the data collection, the project was first prepared by the teacher and students together under the principles of project-based learning, and teaching writing. Then, three consecutive essay writing pretests on different topics were administered to the students to identify their essay writing performance before the intervention. Following the pretests, the intervention, which was a project-based learning essay writing instruction, was delivered for six weeks. During this time in the teaching-learning process, students have completed their tasks, from selecting the project topic to the last stage of evaluating the result and their journey with project-based learning. More specifically, the teacher and students brought different project topics and agreed on one common topic for everyone involved. Hence, the intervention was teaching essay writing through the project "How Can We Make Our Campus Clean?". Students discussed the importance of environmental responsibility and linked it to the role on their campus. Then, in consultation with the teacher, students grouped themselves into four mixed-ability groups and assigned roles within each group. They went through researching and generating ideas, planning solutions and writing proposals, creating awareness campaigns, implementing, and reflecting. They did their project tasks three hours per week, for six weeks. Finally, they evaluated their essays, proposals, and campaign materials for organization, grammar, vocabulary, coherence, and creativity and presented them to the school community. Next to the completion of the project, the participants were given three consecutive essay writing post-tests, which were similar but not identical to the pretests. The post-tests were needed to determine whether the project-based learning made improvements in students’ writing skills.

To assess the pre and post-tests, it was essential to establish clear metrics and use a comprehensive scoring rubric, so the students’ essays were scored using rubrics that focused on writing performance. The writing performance rubric used to evaluate the students’ essays was adapted from the British Council International English Language Testing System (IELTS) Writing Task-2 descriptors (2018) that incorporated Task Achievement, Coherence and Cohesion, Lexical Resource, Grammatical Range, and Accuracy. Two experienced EFL university teachers marked essays independently based on the given criteria. For the raters, inter-rater reliability was calculated at 0.8, implying that the test was reliable. Experts from TEFL checked the validity of the face and content of the tests. By the completion of the post-test, the semi-structured interview was conducted with selected students.

### Data analysis

The study collected qualitative and quantitative data. The quantitative data which were gathered through pre-tests and post-tests were analyzed using One-Way Repeated Measures ANOVA, using the Statistical Package for Social Science (SPSS) version 26 software program. One-Way Repeated Measures ANOVA was used to examine whether there were differences in students’ essay writing skills before and after the intervention. Hence, the study has used descriptive statistics for pretest and post-test scores, the Tests of Within-Subjects Effects, estimates, pairwise comparisons, and multivariate tests. Normality, homogeneity of variance, and sphericity tests were checked to ensure a valid ANOVA analysis. In addition, the semi-structured interview data analysis went through thematic analysis methods. On the other hand, the qualitative data which were collected through interviews were analyzed through narration

## Results

As the data were collected from three consecutive essay writing pretests and another three consecutive essay writing post-tests, this section presents the students’ test results which were analyzed using One Way Repeated Measures ANOVA.

Table 1 shows the descriptive statistics for pretest and post-test scores on the writing skill assessment for EFL learners before and after the intervention. Accordingly, in this study, the mean scores for the three pretests (31.62, 33.23, and 31.31) are relatively close. These scores imply that the baseline writing performance of sample students was nearly similar before the intervention. However, the mean scores for the three post-tests (43.08, 42.62, and 43.62) were higher than the three pretests; this difference indicates an improvement in writing performance after the intervention of project-based learning. This shows how students benefited from the application of project-based learning in their writing classes. The standard deviation scores of the three pretests (3.150, 2.166, and 1.750) indicate that dispersion around the mean scores is relatively low. The standard deviation scores of the three post-tests are 2.465, 3.150, and 3.525. The data indicates that the intervention was successful because participants’ post-test scores significantly improved. Additional qualitative and statistical evaluations could support the validity of these results and assist in enhancing the intervention for more consistent results. Therefore, the data from the descriptive statistics showed how project-based learning significantly improves EFL learners’ writing performance, as evidenced by the increase in mean scores from the pretests to the post-tests.

**Table 1:** Descriptive statistics of students’ essay writing performance.

Tests	Mean	Std. Deviation	N
pretest 1	31.62	3.150	23
pretest 2	33.23	2.166	23
pretest 3	31.31	1.750	23
posttest 1	43.08	2.465	23
posttest 2	42.62	3.150	23
posttest 3	43.62	3.525	23

Tests of Within-Subjects Effects are commonly used in within-subjects designs, where participants are exposed to multiple experimental conditions or repeated measures [30,31]. Table 2 shows the Tests of Within-Subjects Effects, specifically analyzing the effect of project-based learning on EFL learners’ writing skills over time. Based on the data, the significant effect of project-based learning on EFL learners’ writing performance was recorded as there is a significant F-value and a very high partial eta squared value. These within-subjects effects tests support the data from the descriptive statistics and are supported by the data from Sphericity Assumed, Greenhouse-Geisser, Huynh-Feldt, and Lower-bound, which ensure the validity of the results, so the extensive effect size record supports the effectiveness of project-based learning in improving writing performance among EFL students. While the observed power indicates that the tests are reliable, the solid noncentral parameter values indicate the strength of the results. The repeated-measures ANOVA results indicate a substantial effect of time on scores, with evidence of consistent improvement over the measurement points. Strong effect sizes, high power, and consistent significance across sphericity corrections support the robustness of the findings. This suggests that the intervention or progression across the testing periods was highly effective. In general, the results from the test within-subjects effects supported the strength of the evidence in support of the effectiveness of project-based learning in improving EFL learners writing performance significantly and meaningfully.

**Table 2:** Tests of within-subjects effects.

Source		Type III Sum of Squares	df	Mean Square	F	Sig.	Partial Eta Squared	Non-cent. Parameter	Observed Power^a^
Time	Sphericity Assumed	2415.808	5	483.162	82.040	.000	.872	410.202	1.000
	Greenhouse-Geisser	2415.808	2.971	813.263	82.040	.000	.872	243.702	1.000
	Huynh-Feldt	2415.808	4.055	595.726	82.040	.000	.872	332.693	1.000
	Lower-bound	2415.808	1.000	2415.808	82.040	.000	.872	82.040	1.000
Error (time)	Sphericity Assumed	353.359	60	5.889					
	Greenhouse-Geisser	353.359	35.646	9.913					
	Huynh-Feldt	353.359	48.663	7.261					
	Lower-bound	353.359	12.000	29.447					

a. Computed using alpha = .05.

Table 3 presents pairwise comparisons for the effect of project-based learning on EFL learners’ writing skills across different time points. The significant differences between all pretests and all posttests strongly indicate the effectiveness of the intervention. Large mean differences (e.g., −12.308–12.308−12.308 between Pretest 3 and Posttest 3) highlight a substantial impact. The lack of significant differences among posttests indicates that participants maintained a high level of performance across all posttests. This suggests a consistent and lasting effect of the intervention. Therefore, this pairwise comparison table asserts that the writing performance of EFL learners is significantly improved by the application of project-based learning, which can be used as an effective method for improving EFL learners’ writing performance.

**Table 3:** Pairwise comparisons.

(I) time	(J) time	Mean Difference (I-J)	Std. Error	Sig.^b^	95% Confidence Interval for Difference^b^	
					Lower Bound	Upper Bound
Pretest 1	Pretest 2	-1.615	1.077	.160	-3.963	.732
	Pretest 3	.308	.771	.697	-1.373	1.988
	Posttest 1	-11.462*	.730	.000	-13.053	-9.870
	Posttest 2	-11.000*	.884	.000	-12.927	-9.073
	Posttest 3	-12.000*	1.240	.000	-14.702	-9.298
Pretest 2	Pretest 1	1.615	1.077	.160	-.732	3.963
	Pretest 3	1.923*	.746	.024	.297	3.549
	Posttest 1	-9.846*	.999	.000	-12.023	-7.669
	Posttest 2	-9.385*	1.130	.000	-11.846	-6.923
	Posttest 3	-10.385*	1.448	.000	-13.539	-7.230
Pretest 3	Pretest 1	-.308	.771	.697	-1.988	1.373
	Pretest 2	-1.923*	.746	.024	-3.549	-.297
	Posttest 1	-11.769*	.622	.000	-13.124	-10.415
	Posttest 2	-11.308*	.654	.000	-12.733	-9.882
	Posttest 3	-12.308*	.983	.000	-14.450	-10.166
Posttest 1	Pretest 1	11.462*	.730	.000	9.870	13.053
	Pretest 2	9.846*	.999	.000	7.669	12.023
	Pretest 3	11.769*	.622	.000	10.415	13.124
	Posttest 2	.462	.462	.337	-.544	1.467
	Posttest 3	-.538	.971	.590	-2.655	1.578
Posttest 2	Pretest 1	11.000*	.884	.000	9.073	12.927
	Pretest 2	9.385*	1.130	.000	6.923	11.846
	Pretest 3	11.308*	.654	.000	9.882	12.733
	Posttest 1	-.462	.462	.337	-1.467	.544
	Posttest 3	-1.000	1.056	.362	-3.301	1.301
Posttest 3	Pretest 1	12.000*	1.240	.000	9.298	14.702
	Pretest 2	10.385*	1.448	.000	7.230	13.539
	Pretest 3	12.308*	.983	.000	10.166	14.450
	Posttest 1	.538	.971	.590	-1.578	2.655
	Posttest 2	1.000	1.056	.362	-1.301	3.301

*. The mean difference is significant at the 0.05 level. b. Adjustment for multiple comparisons: Least Significant Difference (equivalent to no adjustments).

Table 4 presents the estimates for the effect of project-based learning on EFL learners’ writing skills across different time points. The concept of error is central to estimates, precision, and accuracy. The mean score in Table 4 shows an apparent enhancement of EFL learners’ writing performance across pretests and post-tests, and the mean estimates are reliable with low standard error values. Again, a significant difference between pretest and post-test scores is recorded as the confidence intervals do not overlap. These estimate scores, in general, indicate that EFL learners writing performance improved significantly after the implementation of project-based learning. The low standard error values support this improvement in learners’ writing performance. Therefore, the writing performance of EFL learners is significantly improved after the implementation of project-based learning.

**Table 4:** Estimates.

Tests	Mean	Std. Error	95% Confidence Interval	
			Lower Bound	Upper Bound
Pretest 1	31.615	.874	29.712	33.519
Pretest 2	33.231	.601	31.922	34.540
Pretest 3	31.308	.485	30.250	32.365
Posttest 1	43.077	.684	41.587	44.567
Posttest 2	42.615	.874	40.712	44.519
Posttest 3	43.615	.978	41.485	45.745

Table 5 presents multivariate tests for the effect of PBL on EFL learners’ writing skills across different time points. Accordingly, Pillai’s trace (.979), Wilks’ lambda (.021), Hotelling’s trace (47.448), and Roy’s largest root (47.448) values suggest the strong multivariate effect of time. In addition, a significantly high F-value (75.917) across the tests indicates that the difference is statistically significant. In addition, Table 5 presents the multivariate tests of Partial Eta Squared (.979) and Noncent. Parameter (379.587) indicates a strong effect of project-based learning, and Observed Power (1.000) further indicates the high reliability of the test, which in turn supports the impact of project-based learning on learners’ writing performance. In sum, these findings show that project-based learning effectively enhances EFL learners’ writing performance. The multivariate tests consistently demonstrate that time has a significant effect on scores, with nearly all the variance explained by this factor. These results strongly support the effectiveness of the intervention or progression measured across the time points.

**Table 5:** Multivariate tests.

	Value	F	Hypothesis df	Error df	Sig.	Partial Eta Squared	Noncent. Parameter	Observed Power^b^
Pillai’s trace	.979	75.917^a^	5.000	8.000	.000	.979	379.587	1.000
Wilks’ lambda	.021	75.917^a^	5.000	8.000	.000	.979	379.587	1.000
Hotelling’s trace	47.448	75.917^a^	5.000	8.000	.000	.979	379.587	1.000
Roy’s largest root	47.448	75.917^a^	5.000	8.000	.000	.979	379.587	1.000

Each F tests the multivariate effect of time. These tests are based on the linearly independent pairwise comparisons among the estimated marginal means.

a. Exact statistic.

b. Computed using alpha = 0.05

The data from the semi-structured interview revealed several key themes and insights related to the effect of project-based Learning on EFL learners’ writing skills.

Students’ experiences with project-based learning were one prominent theme that emerged from the interviews. Students consistently highlighted the application of project-based learning, indicating its tremendous significance in writing classes. For example, one student says, "My experience with the project in our writing class has been incredibly interesting and impactful. Unlike other writing assignments that often felt fixed and uninspiring, that project allowed me to explore writing more authentically and engagingly". This implies that project-based learning played an important role in inspiring students to do writing tasks better than conventional teaching methods.

Students’ preference for writing projects over traditional assignments was taken from the interviews was students’ preference for writing projects over traditional assignments. Students highlight their preference for writing projects over traditional assignments, citing reasons such as increased engagement, autonomy, creativity, ownership, and collaboration. A notable example is, "I found working on writing projects to be much more motivating and enjoyable compared to traditional assignments. With projects, I felt a sense of ownership over my writing and a greater investment in the outcome. Traditional assignments sometimes felt like busywork, but projects allowed me to dive deeper into topics and produce writing that I was proud of." This implies that project-based learning made writing interesting which is very important to produce effective writing products.

Common challenges students may encounter in project-based learning was the third theme that was taken from the interview, and students highlighted the common challenges, including time management, generating ideas, incorporating feedback, revising effectively, and staying motivated throughout the writing process. One of the respondents said, "The most challenging aspect of engaging in project-based learning was budgeting my time effectively. There were multiple stages involved in writing projects, from research and planning to drafting and revising. Finding a balance between project-based learning stages and juggling other coursework and commitments required careful planning and organization."

Regarding the impact of project-based learning activities on students’ writing abilities and confidence, students emphasize the positive impact of project-based learning activities on students’ writing abilities and confidence, underscoring improvements in various aspects of writing skills. For instance, one student said, "My participation in the project significantly improved my writing abilities and boosted my confidence in writing. Through engaging in projects, I noticed a marked improvement in my writing skills, including my ability to articulate ideas clearly, use language effectively, and cohesively structure my writing. Moreover, receiving feedback from peers and the teacher during the project helped me identify areas for improvement and refine my writing further, which contributed to my overall growth and confidence." Another student demonstrated how various writing projects in the course influenced students’ writing development by providing opportunities for exploration, experimentation, collaboration, research, and self-reflection. He said, "One writing project that stood out to me was when we were tasked with creating a problem-solving essay on the topic of "How to Make Our Campus Clean." The project required extensive research, critical analysis, and expository writing skills. Through this project, I learned how to effectively structure and organize my essay and use evidence to support my claims. It also helped me develop my research skills and taught me how to synthesize information from multiple sources into a coherent and formal narrative. Overall, this project challenged me to think critically about complex issues and strengthened my ability to communicate my ideas formally in writing." The respondent explained how project-based learning helped him in his essay writing skills, and critical-thinking skills. Hence, it supports the quantitative data explained before.

Finally, students were asked about the impact of their collaboration, which is one aspect of project-based learning, on their writing. Highlighting benefits such as idea generation, feedback exchange, perspective diversity, motivation, and overall improvement in writing quality, they revealed the positive impact of collaboration on their writing. Regarding this idea, a student said, "Working with peers provided me with valuable opportunities for brainstorming, problem-solving, and idea generation, receiving constructive feedback on my writing, identifying areas for improvement, and gaining new perspectives on my work". This response implies that in their projects, students developed their cooperation in a better way which in turn helped them write well-organized essays because while doing their project, students were assigned to different groups so that they shared tasks and completed the project as a group.

In general, these themes that are gained from students’ interviews, paint a nuanced picture of the effect of project-based Learning on EFL learners’ writing and offer valuable insights into its teaching. Project-based learning provided students with opportunities to engage in authentic writing tasks, which enhanced their motivation and engagement in the writing process and led to a significant improvement in the participants’ overall writing skills.

## Discussion

After rigorously analyzing the qualitative and quantitative data, this study confirms the significant contribution of project-based learning to improving the writing skills of EFL students. The findings of the study prove that the application of project-based learning in writing classes enhances EFL learners’ writing performance. A systematic analysis of the pretest and post-test scores was made, and to complement the quantitative research on student writing in project-based learning activities, this study conducted an in-depth qualitative analysis of the interview data. So, the study proved that students showed significant improvements in their writing skills; in addition, they are optimistic about the application of project-based learning in their essay writing class. These improvements can be attributed to several factors. For instance, project-based learning provides students with authentic and meaningful writing tasks, which enhances their motivation and engagement in the writing process; even other studies stated that authentic materials are helpful for language classrooms. In this study, students did a project that employed the situation around them. The project, as it was directly related to their day-to-day life, motivated them to solve and suggest solutions for initial problems. The collaborative nature of project-based learning allowed students to receive feedback from their peers and the teacher. While engaging in the project, students did tasks in pairs and groups. This created an opportunity for the students to generate ideas, gather information, plan and outline drafts, give and receive comments and feedback, and overall improve in writing quality. As they are compatible with the steps of the writing process, the stages involved in project-based learning enabled students to know the subject of writing very well, to research and collect necessary data, to plan and to make outlines, to make many drafts, to revise the content and organization of their writing material before submission. Through these procedures, project-based learning provided opportunities for students to apply various writing strategies and techniques, further developing their writing proficiency, as well as self-evaluation and peer evaluation techniques.

According to the quantitative data, a significant increase in performance is demonstrated by statistical comparisons between pre- and post-tests, as well as higher writing scores on standardized rubrics that assess certain writing components. The qualitative data from student interviews, showed a more positive perception of students towards project-based learning when it came to teaching writing skills. Students’ qualitative reports of feeling more competent and confident in their writing frequently correspond with an increase in quantitative scores.

These findings align with previous research on the effects of project-based learning on EFL learners’ writing skills. For instance, [32] found that project-based learning significantly improved students’ writing skills in German as a foreign language classroom. Similarly, [33] reported that project-based learning had a positive impact on students’ writing performance in a Japanese language learning context. These studies are worth reporting the change in students’ writing performance because of the application of project-based learning on two different intact groups. They also examined the enhancement of EFL learners writing skills through project-based learning in a nutshell rather than indicating the specific genre of writing, which showed improvement as a result of the intervention. Hence, the current study exposed the effects of project-based learning writing instruction with particular emphasis on expository writing on EFL learner’s writing skills. The study supported the existing literature by providing additional evidence on the effects of project-based learning on EFL learners’ writing performance; moreover, the study could assist the field of ELT as it provided the effect of project-based learning on students writing performance in the local context, where project-based learning is a new but essential feature of ELT classrooms.

## Conclusions

The study aimed to investigate the effect of project-based learning on EFL learners’ writing performance. The findings of this study revealed that project-based learning enhances EFL learners writing performance in several ways. Project-based learning provides opportunities for EFL learners to engage in authentic writing tasks that simulate real-world contexts and allow for the integration of various language skills, including reading, writing, speaking, and listening. It also promotes collaborative writing experiences, where learners work together to plan, draft, and revise written texts. It also provides opportunities for EFL learners to write for authentic audiences and purposes, increasing the relevance and motivation of writing tasks. Project-based learning encourages iterative writing processes, where learners receive feedback from peers and instructors and revise their work accordingly; it facilitates ongoing formative assessment of EFL learners’ writing skills throughout the project duration. Therefore, taking all the findings into account, project-based learning is another means of fostering EFL learner’s writing skills. Through its authentic writing tasks, its ability to integrate various language skills, its interactive and collaborative nature, and its emphasis on ongoing formative assessment, project-based learning enhances writing skills in EFL classrooms.

### Implications

This study found the positive effects of project-based learning on EFL students’ writing skills. Hence, by showing how project-based learning complements and encourages active, student-centred learning in the context of EFL writing, the study advances our understanding of constructivist learning theories. Beyond its theoretical contributions, the research offers a methodical framework for using project-based learning to enhance writing performance, along with techniques for task creation, scaffolding, and evaluation.

The findings of the study hold several pedagogical implications that can significantly influence teaching practices and curriculum design in EFL contexts. Therefore, EFL educators can design projects that require learners to produce written artefacts, which could foster the development of practical writing skills applicable beyond the classroom and require learners to research, analyze information, collaborate with peers, and present their findings, which in return promotes holistic language development while focusing on writing skills. Teachers can structure group projects that require learners to allocate roles and responsibilities, negotiate ideas, and co-create written documents, which could enhance collaboration and peer learning in the writing process. They can incorporate opportunities that would enhance the quality of learners’ writing by fostering a culture of feedback and revision. Projects could also involve communication with external stakeholders, such as community members, experts, or peers from other classrooms, which could create meaningful contexts for writing. Moreover, EFL teachers should be trained on how to efficiently integrate project-based learning into their teaching methodologies, maximizing its potential to optimize students’ language learning results.

### Limitations

The current study filled some project-based learning and writing instruction research gaps, but it has some limitations. The relatively small sample size and the fact that all participants were English-major students from a single academic institution in Ethiopia may limit the generalizability of the findings to other populations and EFL contexts. Furthermore, the study focused solely on the impact of project-based learning on EFL learners’ essay writing proficiency, excluding other critical language skills such as reading, speaking, and listening. In addition, the study was conducted for six weeks, which may not be sufficient to fully understand the long-term effects of the intervention on students’ essay-writing skills. One further limitation worth noting is the presence of a one-group pretest-posttest design. Although it allows for the observation of changes in participants’ performance over time, it lacks a control group or comparison group, and there was no randomization. Addressing these issues will significantly improve ELT through project-based learning, ensuring that it remains a relevant, innovative, and effective approach to English learning and teaching.

## Supporting information

S1 Raw data (XLSX)
